# The IGCA staging system is more accurate than AJCC7 system in stratifying survival of patients with gastric cancer in stage III

**DOI:** 10.1186/s12885-017-3235-3

**Published:** 2017-03-31

**Authors:** Ping Shu, Jing Qin, Kuntang Shen, Weidong Chen, Fenglin Liu, Yong Fang, Xuefei Wang, Hongshan Wang, Zhenbin Shen, Yihong Sun, Xinyu Qin

**Affiliations:** Department of General Surgery, Zhongshan Hospital, Fudan University, No. 180 Fenglin Road, Shanghai, 200032 People’s Republic of China

**Keywords:** Gastric cancer, Lymph node, TNM staging, Prognosis, Overall survival

## Abstract

**Background:**

A new staging system recently proposed by the IGCA has demonstrated a better capacity of stratifying different prognoses for gastric cancer than the 7th edition AJCC staging system (AJCC7). The aim of this study was to evaluate the efficacy of the IGCA system in Chinese patients.

**Methods:**

Medical records of patients with gastric cancer who received curative surgery in our center from January 2003 to December 2011 were reviewed retrospectively. All the lesions were staged according to both AJCC7 and IGCA staging systems. Overall survival (OS) of the patients was used as the observation endpoint.

**Results:**

One thousand five hundred twenty-six cases were included in this study. By comparing the AJCC7 system with the IGCA systems, 395 cases were stratified into different stages, most of which were in stage III. The IGCA system could better stratify stage IIIB and IIIC patients (5-year OS, 38.1% vs. 29.0%; *P* = 0.005) than the AJCC7 system (5-year OS, 38.2% vs. 35.9%; *P* = 0.148). T3N3bM0, T4aN2M0 and T4aN3bM0 made up 97.5% (385/395) of the stage shift. T3N3bM0, which was stratified to stage IIIB in the AJCC7 system, showed a significant poorer prognosis than T4aN2M0 and T4aN3aM0, which were staged to IIIB and IIIC in the same system. The improper staging was revised in the IGCA staging system.

**Conclusions:**

The IGCA staging system can stratify stage III gastric cancer patients more properly than the AJCC7 system.

**Electronic supplementary material:**

The online version of this article (doi:10.1186/s12885-017-3235-3) contains supplementary material, which is available to authorized users.

## Background

Gastric cancer is the fourth most common cancer and the second leading cause of cancer-related death [1, 2]. Lymph node (LN) metastasis is the most common metastatic pattern and the most important factor that impacts the prognosis of gastric cancer. However, there is no real consensus over the definition of LN staging. The Japanese Classification of Gastric Carcinoma (JCGC) used to assess the metastatic status of LN according to the anatomical distribution and this classification was widely applied in China because it could properly depict the extent of lymph node removal of surgery. However, many studies argued that the numeric LN staging system proposed by the American Joint Committee on Cancer (AJCC) TNM staging system was simpler and more practical which demonstrated a better prognostic prediction than the anatomical LN staging pattern [3].

The latest edition of the AJCC is the seventh edition (AJCC7) published in 2010, which can more precisely predict the prognosis of gastric cancer after curative surgery by revising the cutoffs of metastatic lymph node counts in the previous edition [4, 5]. In this edition, previous N1 stage (metastasis in 1-6 regional LN) is divided into N1 (metastasis in 1-2 regional lymph nodes) and N2 (metastasis in 3-6 regional lymph nodes). Besides, N3 stage is sub-grouped to N3a (metastasis in 7-15 regional LN) and N3b (metastasis in more than 15 regional LN). According to the existing literature [6, 7], a much better prognosis was observed in patients with N3a stage than those with N3b. However, the AJCC7 gastric staging system fails to incorporate N3a and N3b into any stage group, which would impact the prognostic prediction of advanced diseases, especially for patients with the N3 diseases.

Recently, International Gastric Cancer Association (IGCA) has proposed a new staging system for gastric cancer. This system shares the same TNM classification with the AJCC7 system but introduces pN3a and pN3b into staging. In this system, all resectable lesions are also stratified into seven groups from IA to IIIC as is the cases with the AJCC7 system [7] and each group is classified according to the number of deaths during five-year period after surgery. The aim of this study was to evaluate the suitability of the IGCA staging system for patients with gastric cancer in China.

## Methods

All medical records of gastric cancer patients who received curative surgery in our center from January 2003 to December 2011 were reviewed retrospectively. The criteria for eligibility were histologically proven gastric adenocarcinoma and R0 resection. Patients with M1 lesions (para-aortic LN, hepatic, peritoneal, or other distant metastases) were excluded from this study. Patients who received neoadjuvant therapy were also excluded, knowing that it may affect the assessments of the resected specimen and lead to incorrect staging. Demographic data, clinical features, treatment methods and pathological findings were investigated based on the medical records. Each lesion was classified by TNM classification, and then stratified according to the AJCC7 and IGCA staging systems independently.

Follow-up was carried out in the outpatient department and/or through telephone interviews. The observation endpoint was overall survival (OS). OS was defined as the duration from surgery to the last follow-up or patient death. The Kaplan-Meier method and log-rank test were used to compare OS within patients of different stages. All tests were two-tailed and *P* < 0.05 was considered statistically significant. All statistical analyses were carried out using SPSS 17.0.

## Results

**Fig. 1:**
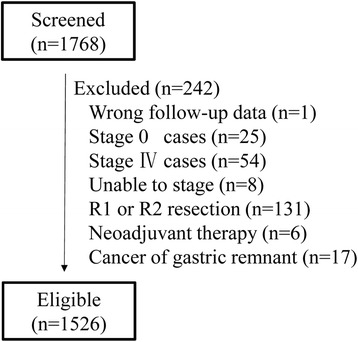
List of the ineligible reasons in this study

**Fig. 2:**
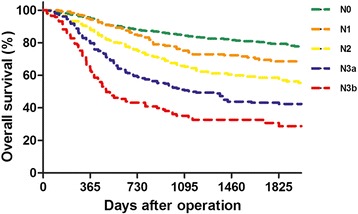
The distribution of OS curves of N stages

Between January 2003 and December 2011, 1768 consecutive cases were collected, of which 242 were deemed ineligible for the reasons listed in Fig. 1. The clinical and pathologic features of the included patients are listed in Table 1. They included 1024 men and 502 women with a median age of 63 (range 22-95) years at the time of surgery. Patients with early cancers (pT1 stage) only accounted for 19.5%. The number of retrieved LN was 23.60 ± 10.59. The prognosis of N3a subgroup (7–15 involved lymph nodes) was significantly better than N3b (>15 involved LN) (5-year OS, 42.4% vs. 28.7%, *P* < 0.001, Fig. 2).

**Table 1:** Characteristics of included patients

Total number	1526
Age (median, range)	63 (22-95)
Sex	
Male	1024 (67.8%)
Female	502 (32.2%)
Location of lesion	
Upper	251 (16.4%)
Middle	309 (20.2%)
Lower	935 (61.3%)
entire	31 (2.0%)
pT stage	
T1	297 (19.5%)
T2	197 (12.9%)
T3	343 (22.5%)
T4a	670 (43.9%)
T4b	19 (1.2%)
LN number retrieved (mean ± SD)	23.60 ± 10.59
pN stage	
N0	554 (36.3%)
N1	242 (15.9%)
N2	273 (17.9%)
N3a	312 (20.4%)
N3b	145 (9.5%)
Surgical procedure	
Distal gastrectomy	1109 (72.7%)
Proximal gastrectomy	193 (12.6%)
Total gastrectomy	224 (14.7%)

**Fig. 3:**
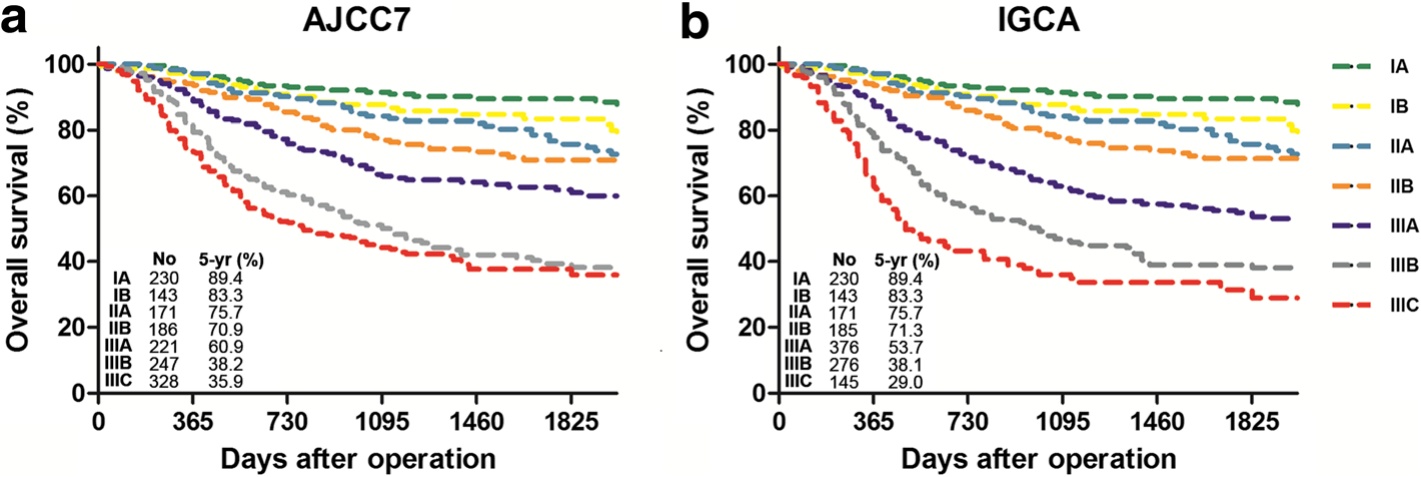
The distribution of OS curves of the different stages grouped by **a**.AJCC7 staging system; **b**. IGCA staging system

According to the AJCC7 and the IGCA systems, 395 cases were stratified into different stages, and most of them fell in stage III (Table 2). Only one case of T1N3b, which was stratified into stage IIB in AJCC7 shifted to stage IIIB in the IGCA system. So the distribution of patients in stage I and II was quite consistent between the two systems. The survival curves showed dissimilarity in stage III patients of the two systems (Fig. 3). Both AJCC7 and IGCA systems demonstrated a much better OS for IIIA patients than that for IIIB or IIIC patients (*P* < 0.001, Fig. 3a, b). However, the IGCA system could better stratify stage IIIB and IIIC patients (5-year OS, 38.1% vs. 29.0%; *P* = 0.005, Fig. 3b) than the AJCC7 system (5-year OS, 38.2% vs. 35.9%; *P* = 0.148, Fig. 3a).

**Table 2:** Seven groups stratified differently in the two staging systems

		N0 (0)	N1 (1-2)	N2 (3-6)	N3a (7-15)	N3b (>15)
T1	AJCC7	IA (*n* = 230)	IB (*n* = 43)	IIA (*n* = 16)	IIB (*n* = 8)	
	IGCA				IIB (*n* = 7)	IIIB (*n* = 1)
T2	AJCC7	IB (*n* = 100)	IIA (*n* = 44)	IIB (*n* = 29)	IIIA (*n* = 24)	
	IGCA				IIIA (*n* = 21)	IIIB (*n* = 3)
T3	AJCC7	IIA (*n* = 111)	IIB (*n* = 51)	IIIA (*n* = 94)	IIIB (*n* = 87)	
	IGCA				IIIB (*n* = 62)	IIIC (*n* = 25)
T4a	AJCC7	IIB (*n* = 98)	IIIA (*n* = 103)	IIIB (*n* = 153)	IIIC(*n* = 317)	
	IGCA			IIIA (*n* = 153)	IIIB (*n* = 207)	IIIC (*n* = 110)
T4b	AJCC7	IIIB (*n* = 5)	IIIB (*n* = 2)	IIIC (*n* = 1)	IIIC (*n* = 10)	
	IGCA	IIIA (*n* = 5)		IIIB (*n* = 1)	IIIC(*n* = 6)	IIIC (*n* = 4)

Only 332 patients in this cohort with advanced diseases had adjuvant chemotherapy in our center. For these patients, the IGCA system also better stratified the prognoses of patients in different stages than the AJCC7 system (Additional file 1: Figure S1).

**Fig. 4:**
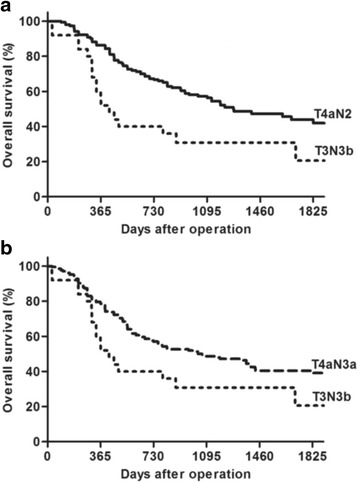
Comparison of the survival of T3N3b, T4aN2 and T4aN3a. **a**. T3N3b and T4aN2, which were both grouped to III B in AJCC7, were indicated a different survival, *P* = 0.003. **b**. T3N3b still had a poorer survival than T4aN3a (III C in AJCC7), *P* = 0.030. The 5-year survival rate of T3N3b, T4aN2 and T4aN3a was 20.6%, 43.9% and 39.1% respectively

Most cases with a stage shift between the two systems were in the following three groups: T3N3b, T4aN2 and T4aN3a (Table 2). The patients in T3N3b had a significant shorter OS than those in T4aN2 (*P* = 0.003, Fig. 4a) and T4aN3a (*P* = 0.030, Fig. 4b).

## Discussion

The TNM stage is the most important factor used to instruct treatment strategies in patients with gastric cancer and indicate the prognosis. The AJCC7 TNM classification is the latest staging system for gastric cancer and contains major modifications compared with the previous editions. In this edition, N1 stage in the 6th edition is divided into N1 (metastasis in 1-2 regional LN) and N2 (metastasis in 3-6 regional LN) based on the different prognoses. N2 (metastasis in 7-15 regional LN) and N3 (metastasis in more than 15 regional LN) in the 6th edition are defined as N3a and N3b in the AJCC7. It was found in this study that survival was much better in patients with N3a stage than that in patients with N3b stage, which is consistent with the existing literature [6, 8, 9]. This result was also recognized in the AJCC7 staging system. However, N3a and N3b were still grouped together as N3 for TNM staging, which could impact the proper prediction of the stage-based prognosis.

In contrast, the IGCA took N3a and N3b separately and established a new staging system in order to better stratify gastric cancer patients with different prognoses. In this staging system, every N3 stage is subdivided into N3a and N3b, whereby patients are divided into 25 TNM subgroups (Table 2) when M1 was excluded [7]. However, compared with the AJCC7 staging system, only seven TNM subgroups (T1N3b, T2N3b, T3N3b, T4aN2, T4aN3a, T4bN0 and T4bN2) have stage shift in IGCA system. T2N3b, T3N3b, T4aN2, T4aN3a, T4bN0 and T4bN2 all fall in stage III in both AJCC7 and IGCA systems but classified into different groups. Although T1N3b shifts from stage IIB in the AJCC7 system to stage IIIB in the IGCA system, there are rare patients in this subgroup. The IGCA system mostly redistributes patients in the three groups (IIIA, IIIB and IIIC) of stage III.

It was found in this study that the AJCC7 system excellently separated the survival curves of stage IIIA from IIIB and IIIC, but failed to discriminate the prognosis of patients in stage IIIB and stage IIIC. The IGCA systems precisely stratified the survival probabilities of patients in IIIA, IIIB and IIIC. This change is obviously due to the stage shift of the seven TNM subgroups mentioned above. 395 cases were included in the seven groups and most of them were stageIII in the AJCC7 or IGCA systems except for one T1N3b case. Since T1N3b (*n* = 1), T2N3b (*n* = 3), T4bN0 (*n* = 5) and T4bN2 (*n* = 1) contained very few cases, the stage shift of the other three subgroups (T3N3b, T4aN2 and T4aN3a) played a leading role on the change of survival curves in stage III. The results of this study indicate that the three subgroups are not properly staged in the AJCC7 systems. T3N3b and T4aN2 are both in IIIB, but the 5-year survival rate of T4aN2 (43.9%) was much better than that of T3N3b (20.6%). Even T4aN3a in stage IIIC had a better survival than T3N3b. The improper staging is revised in the IGCA staging system. The 5-year survival rate of T4aN3a was 39.1%, which perfectly matched that of stage IIIB (38.1%) in the IGCA system. As a matter of fact, the 5-year survival rate of T3N3b was even poorer than stage IIIC disease. Whether this subgroup, together with T4N3b [6], should be considered as stage IV diseases needs further assessment [10–12].

As mentioned above, the IGCA system shows almost no revision of stage I and II in AJCC7, suggesting that the IGCA staging system does not seem to make up for the defects of AJCC7 on the earlier stages of the disease. When the IGCA staging system is used, more regional LN should be harvested, for less than a minimum number of 16 retrieved LN could cause stage migration by inaccurate LN staging. Besides, an improved survival outcome was reported to be associated with more lymph node harvested (>15) [13–15]. However, the threshold for the harvested LN counts needs to be further studied.

This retrospective study has certain limitations. Firstly, although all the patients in this cohort underwent surgery in our center, many of them did not receive subsequent standard adjuvant therapy here owing to their different sources and economic reasons, which might impact the prognostic assessment. We only analyzed the data of patients who received adjuvant chemotherapy in our own center. The IGCA staging system still showed a better performance in prognostic stratification as indicated in Additional file 1: Figure S1. Secondly, according to the 6th AJCC staging systems, the lesions in esophagogastric junction (EGJ) were not distinguished from those in the upper part of the stomach in this cohort. Although EGJ tumors were recommended to be staged as esophageal cancers [16], some current studies had indicated that the adenocarcinoma of EGJ (Siewert II and Siewert III) showed similar clinical and pathological characteristics to the disease derived from stomach and should be considered as gastric cancer [17, 18].

## Conclusions

In summary, after taking pN3a and pN3b as separate groups, the IGCA system indicates a dissimilarity of survival curves in stage III patients with comparison to AJCC7 system. The result of the present study seems to indicate that the IGCA system is more accurate than the AJCC7 system in stratifying survival of patients with gastric cancer in stage III.

## Additional file

Additional file 1: Figure: S1. The survival distributions of 332 patients who received adjuvant chemotherapy in our own center. a The survival distributions of different stages Grouped by AJCC7 staging system. It was unable to distinguish the OS difference between III B and III C diseases (*P* = 0.958); b The survival distributions of different stages Grouped by IGCA staging system. The survival of III B and III C diseases were perfectly stratified (*P* = 0.003). (TIFF 5298 kb)

## Abbreviations

AJCC: American joint committee on cancer
EGJ: Esophagogastric junction
IGCA: International gastric cancer association
JCGC: Japanese classification of gastric carcinoma
LN: Lymph node
OS: Overall survival
