# Supplementary figures and images for: The IGCA staging system is more accurate than AJCC7 system in stratifying survival of patients with gastric cancer in stage III
Source: BMC Cancer. 2017 Mar 31;17:238. doi: 10.1186/s12885-017-3235-3 (PMC5374584; doi:10.1186/s12885-017-3235-3)

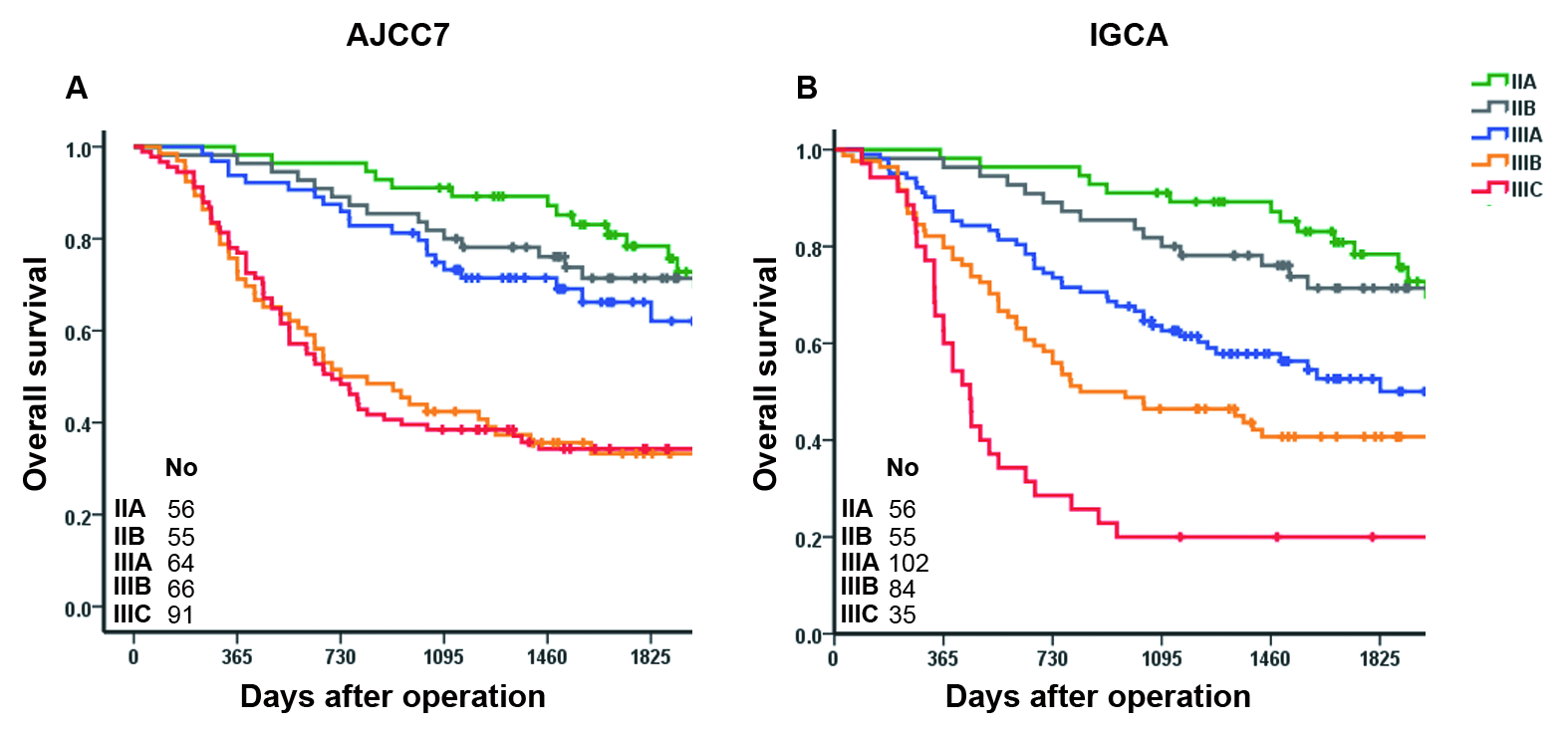

Supplement: Additional file 1: Figure: S1. — The survival distributions of 332 patients who received adjuvant chemotherapy in our own center. a The survival distributions of different stages Grouped by AJCC7 staging system. It was unable to distinguish the OS difference between III B and III C diseases (P = 0.958); b The survival distributions of different stages Grouped by IGCA staging system. The survival of III B and III C diseases were perfectly stratified (P = 0.003). (TIFF 5298 kb) [file 12885_2017_3235_MOESM1_ESM.tif]
